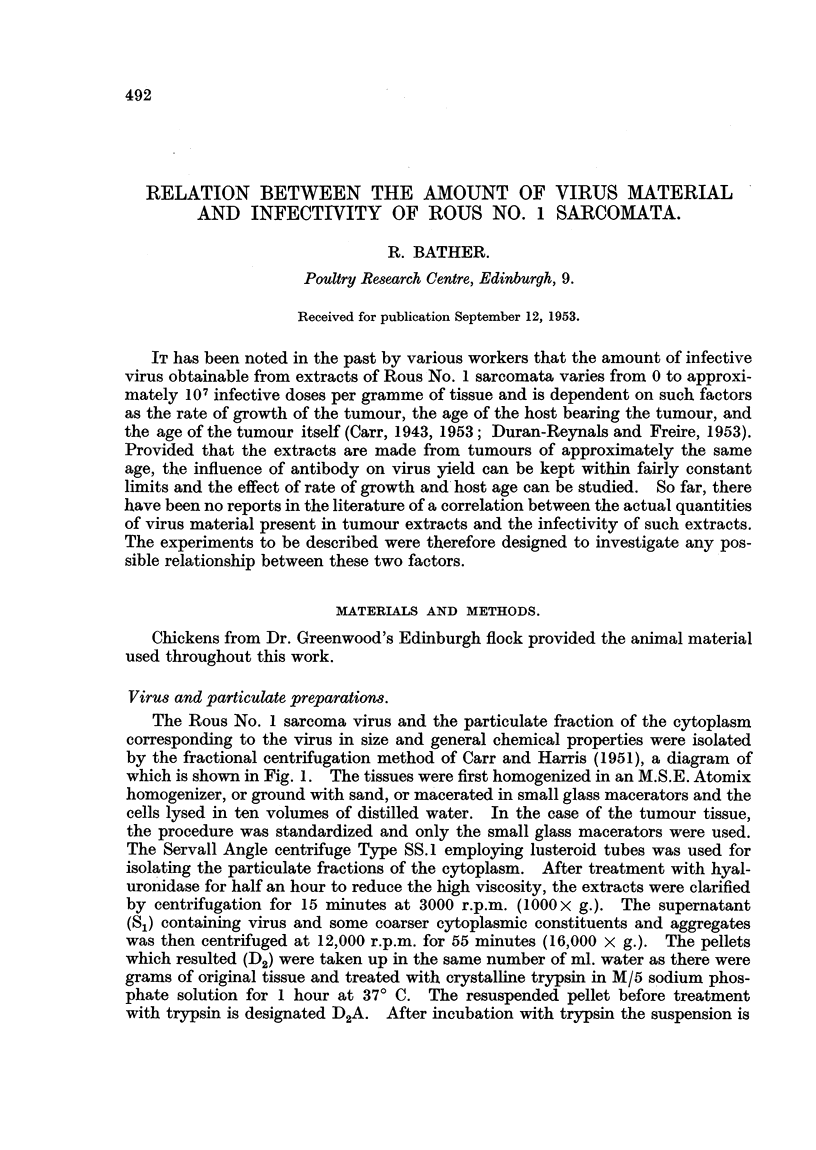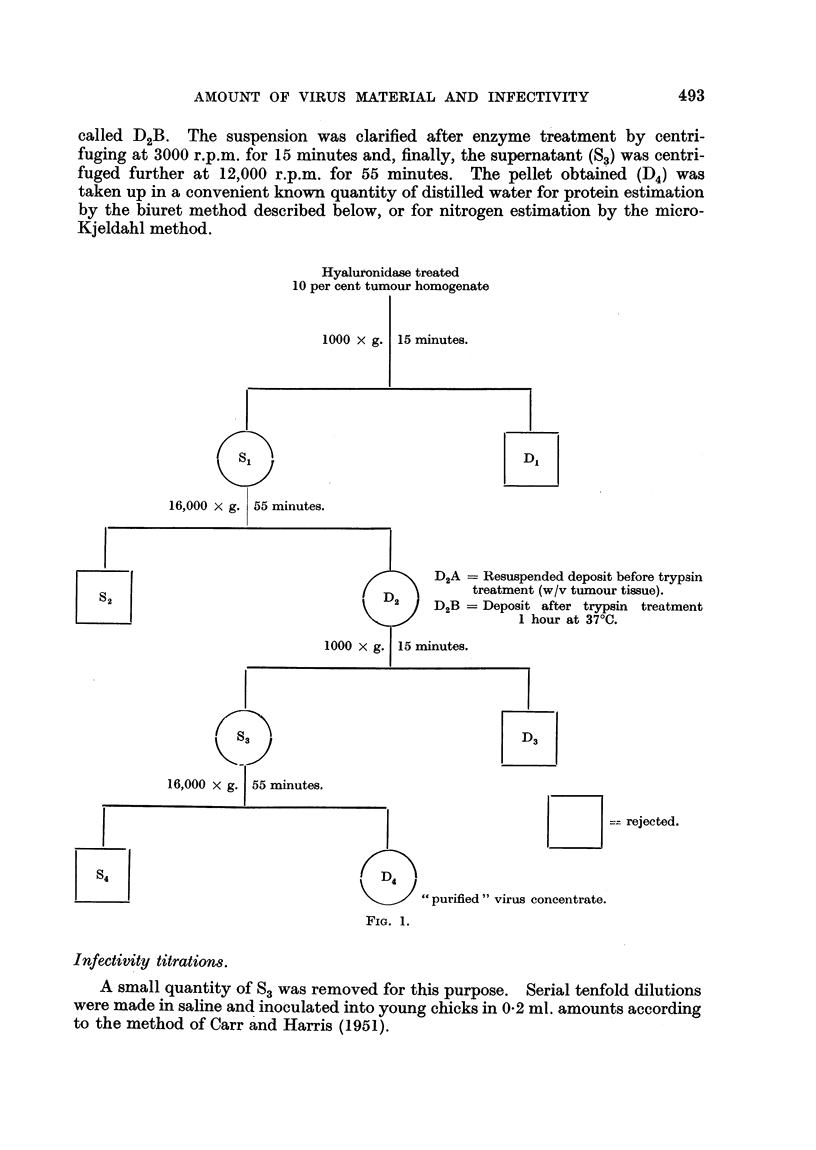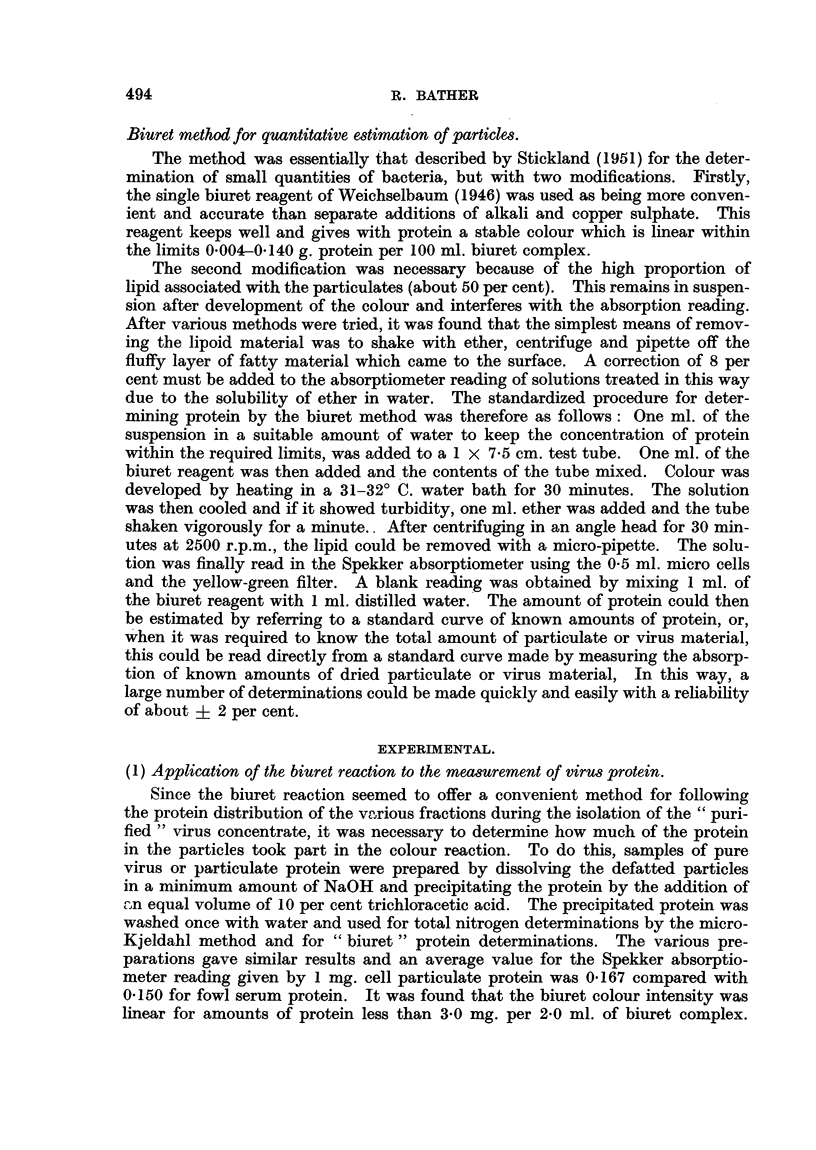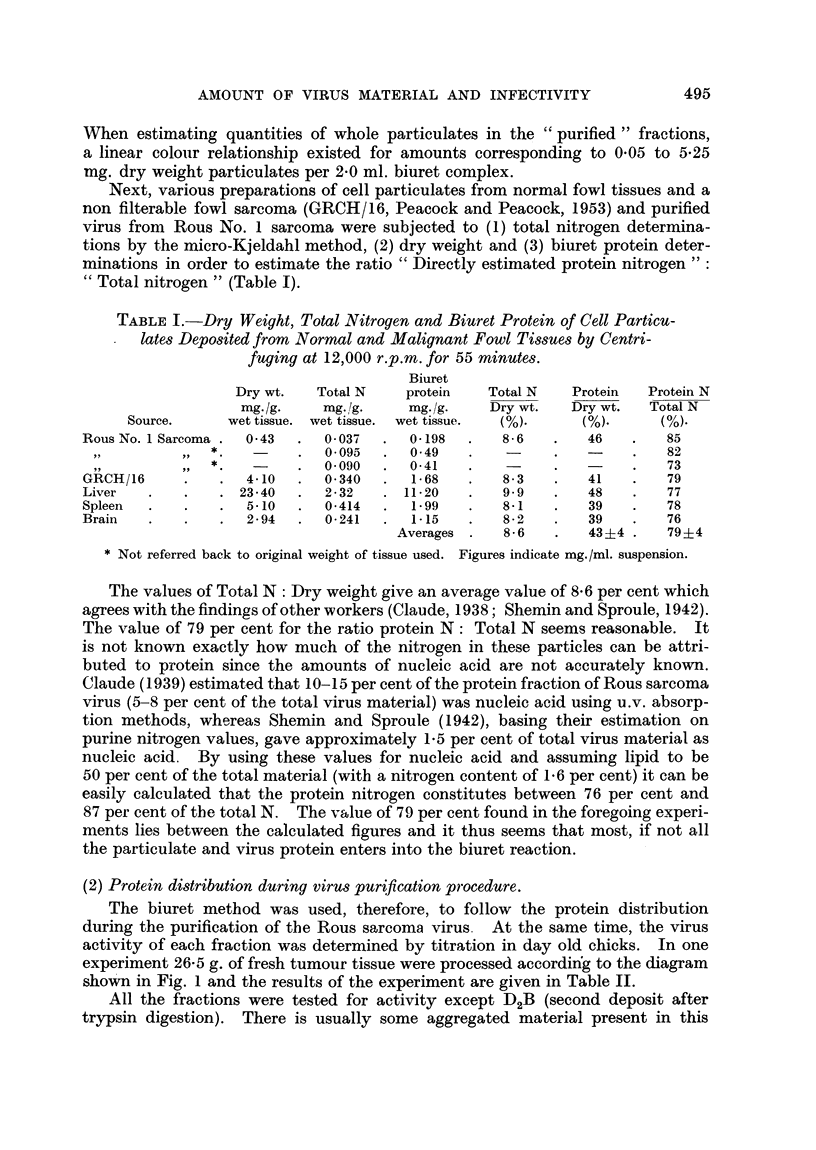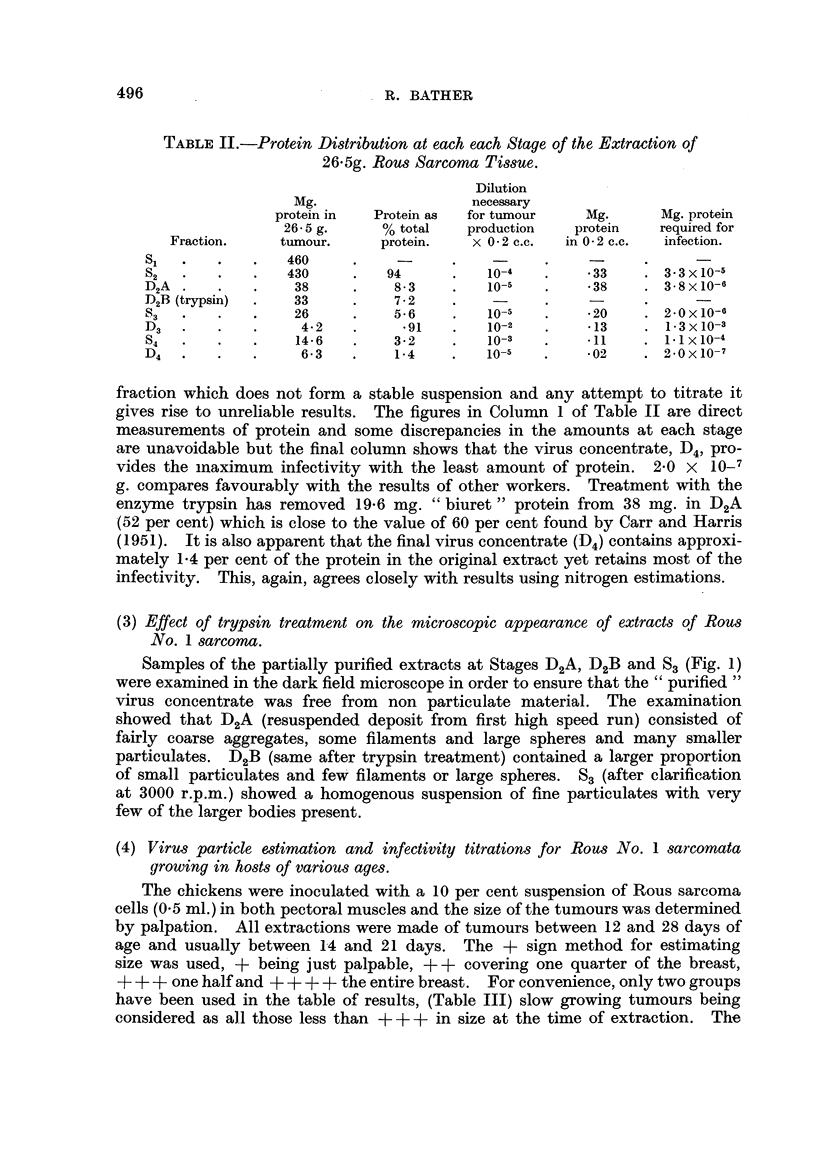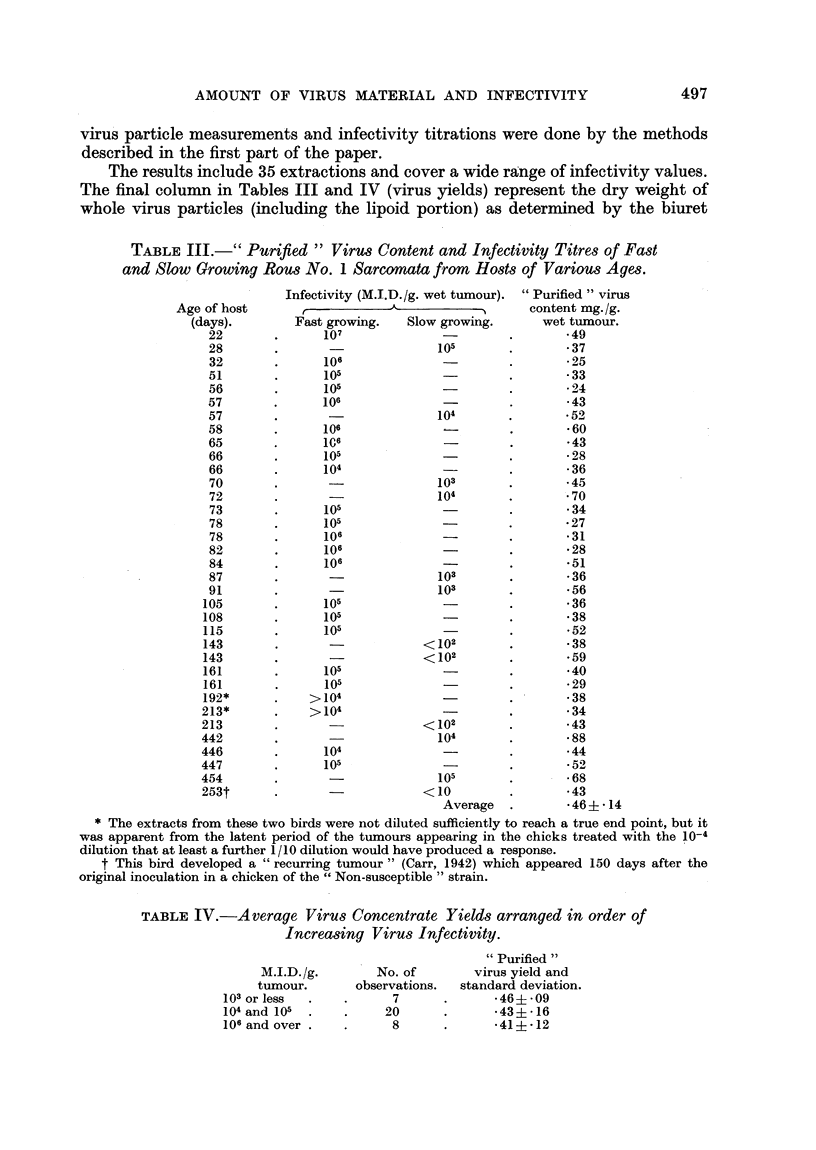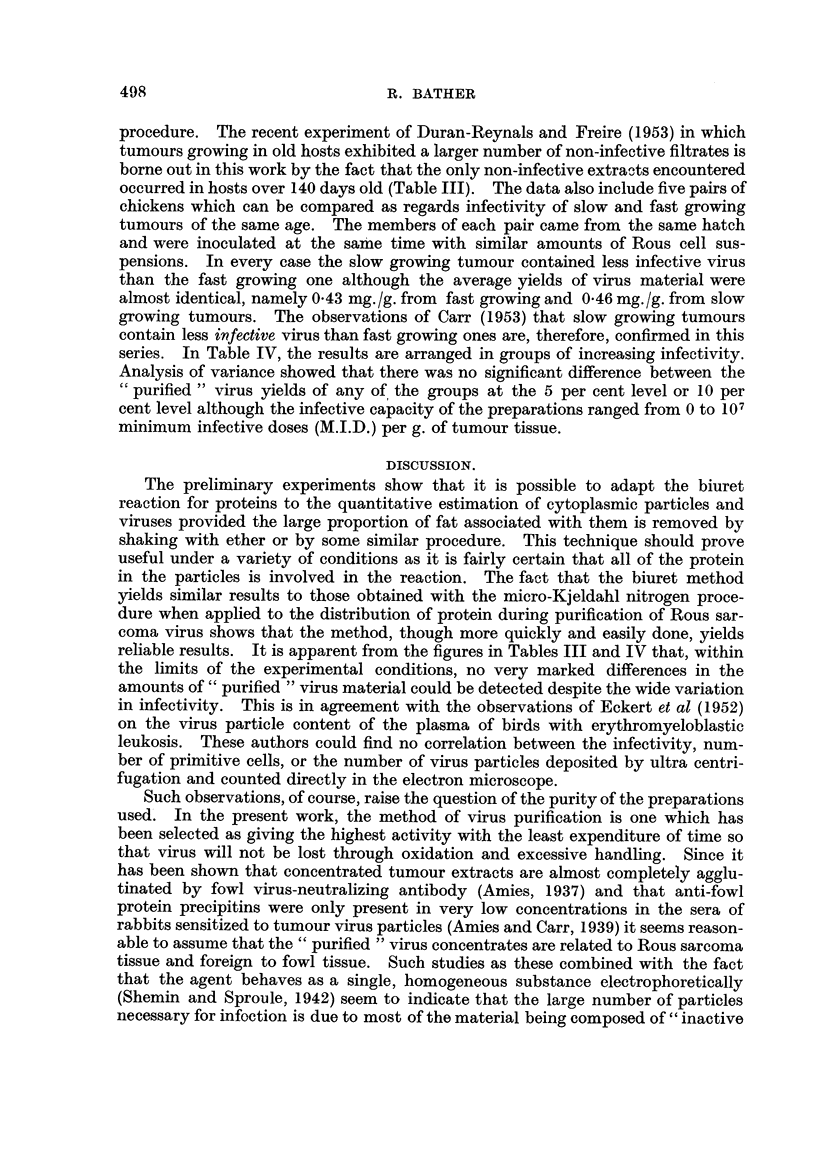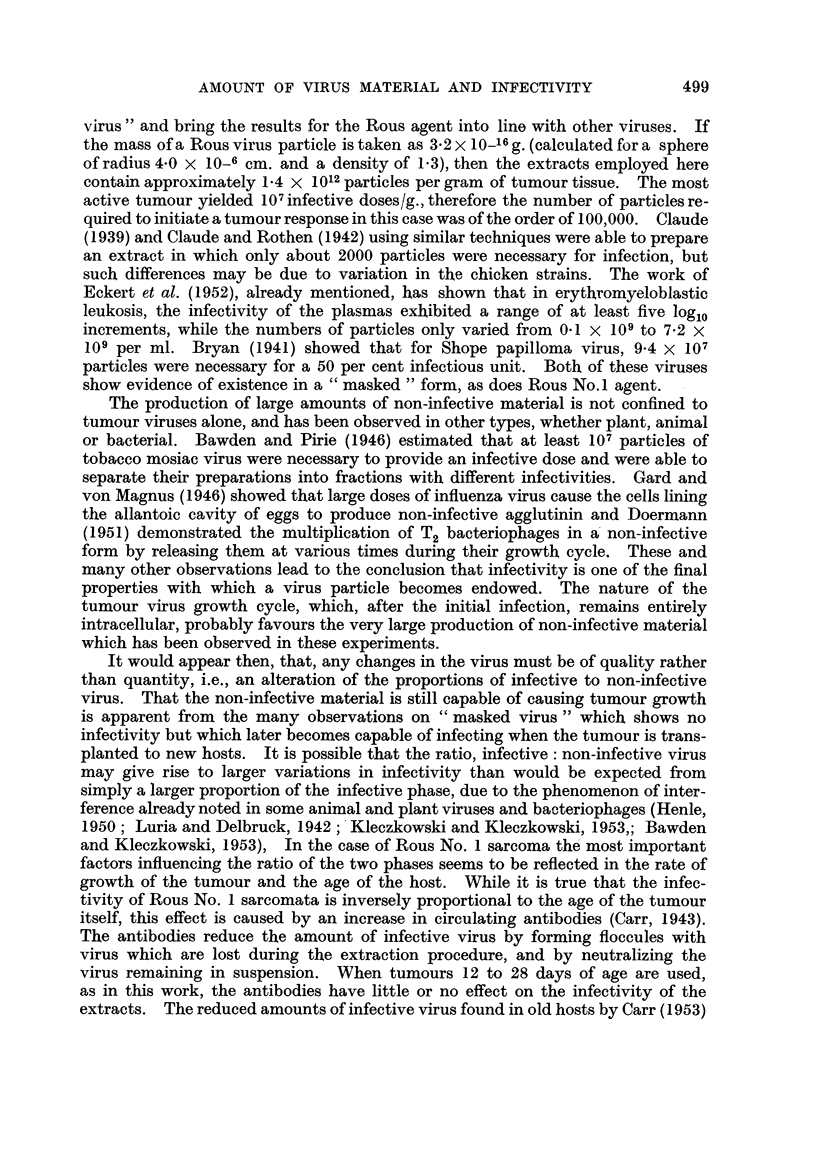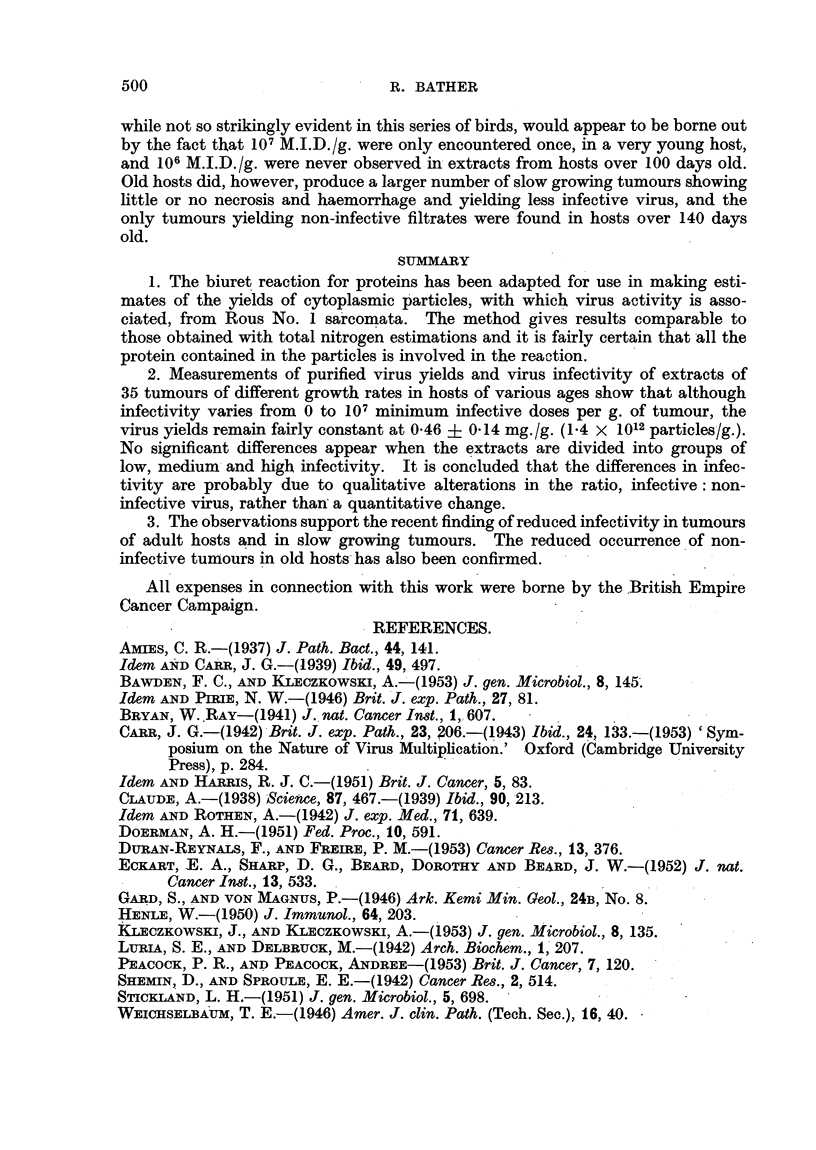# Relation between the Amount of Virus Material and Infectivity of Rous No. 1 Sarcomata

**DOI:** 10.1038/bjc.1953.52

**Published:** 1953-12

**Authors:** R. Bather


### RELATION BETWEEN THE AMOUNT OF VIRUS MATERIAL AND INFECTIVITY OF ROUS NO. 1 SARCOMATA.

R. BATHER.

*Poultry Research Centre, Edinburgh,* 9.

Received for publication September 12, 1953.

IT has been noted in the past by various workers that the amount of infective virus obtainable from extracts of Rous No. 1 sarcomata varies from 0 to approximately $10^7$ infective doses per gramme of tissue and is dependent on such factors as the rate of growth of the tumour, the age of the host bearing the tumour, and the age of the tumour itself (Carr, 1943, 1953; Duran-Reynals and Freire, 1953). Provided that the extracts are made from tumours of approximately the same age, the influence of antibody on virus yield can be kept within fairly constant limits and the effect of rate of growth and host age can be studied. So far, there have been no reports in the literature of a correlation between the actual quantities of virus material present in tumour extracts and the infectivity of such extracts. The experiments to be described were therefore designed to investigate any possible relationship between these two factors.

### MATERIALS AND METHODS.

Chickens from Dr. Greenwood's Edinburgh flock provided the animal material used throughout this work.

### *Virus and particulate preparations.*

The Rous No. 1 sarcoma virus and the particulate fraction of the cytoplasm corresponding to the virus in size and general chemical properties were isolated by the fractional centrifugation method of Carr and Harris (1951), a diagram of which is shown in Fig. 1. The tissues were first homogenized in an M.S.E. Atomix homogenizer, or ground with sand, or macerated in small glass macerators and the cells lysed in ten volumes of distilled water. In the case of the tumour tissue, the procedure was standardized and only the small glass macerators were used. The Servall Angle centrifuge Type SS.1 employing lusteroid tubes was used for isolating the particulate fractions of the cytoplasm. After treatment with hyaluronidase for half an hour to reduce the high viscosity, the extracts were clarified by centrifugation for 15 minutes at 3000 r.p.m. (1000$\times$ g.). The supernatant ($S_1$) containing virus and some coarser cytoplasmic constituents and aggregates was then centrifuged at 12,000 r.p.m. for 55 minutes (16,000 $\times$ g.). The pellets which resulted ($D_2$) were taken up in the same number of ml. water as there were grams of original tissue and treated with crystalline trypsin in M/5 sodium phosphate solution for 1 hour at 37° C. The resuspended pellet before treatment with trypsin is designated $D_2A$. After incubation with trypsin the suspension is called $D_2B$. The suspension was clarified after enzyme treatment by centrifuging at 3000 r.p.m. for 15 minutes and, finally, the supernatant ($S_3$) was centrifuged further at 12,000 r.p.m. for 55 minutes. The pellet obtained ($D_4$) was taken up in a convenient known quantity of distilled water for protein estimation by the biuret method described below, or for nitrogen estimation by the micro-Kjeldahl method.

FIG. 1.

*Infectivity titrations.*

A small quantity of $S_3$ was removed for this purpose. Serial tenfold dilutions were made in saline and inoculated into young chicks in 0·2 ml. amounts according to the method of Carr and Harris (1951).

*Biuret method for quantitative estimation of particles.*

The method was essentially that described by Stickland (1951) for the determination of small quantities of bacteria, but with two modifications. Firstly, the single biuret reagent of Weichselbaum (1946) was used as being more convenient and accurate than separate additions of alkali and copper sulphate. This reagent keeps well and gives with protein a stable colour which is linear within the limits 0·004–0·140 g. protein per 100 ml. biuret complex.

The second modification was necessary because of the high proportion of lipid associated with the particulates (about 50 per cent). This remains in suspension after development of the colour and interferes with the absorption reading. After various methods were tried, it was found that the simplest means of removing the lipoid material was to shake with ether, centrifuge and pipette off the fluffy layer of fatty material which came to the surface. A correction of 8 per cent must be added to the absorptiometer reading of solutions treated in this way due to the solubility of ether in water. The standardized procedure for determining protein by the biuret method was therefore as follows : One ml. of the suspension in a suitable amount of water to keep the concentration of protein within the required limits, was added to a 1 × 7·5 cm. test tube. One ml. of the biuret reagent was then added and the contents of the tube mixed. Colour was developed by heating in a 31–32° C. water bath for 30 minutes. The solution was then cooled and if it showed turbidity, one ml. ether was added and the tube shaken vigorously for a minute.. After centrifuging in an angle head for 30 minutes at 2500 r.p.m., the lipid could be removed with a micro-pipette. The solution was finally read in the Spekker absorptiometer using the 0·5 ml. micro cells and the yellow-green filter. A blank reading was obtained by mixing 1 ml. of the biuret reagent with 1 ml. distilled water. The amount of protein could then be estimated by referring to a standard curve of known amounts of protein, or, when it was required to know the total amount of particulate or virus material, this could be read directly from a standard curve made by measuring the absorption of known amounts of dried particulate or virus material, In this way, a large number of determinations could be made quickly and easily with a reliability of about ± 2 per cent.

### EXPERIMENTAL.

(1) *Application of the biuret reaction to the measurement of virus protein.*

Since the biuret reaction seemed to offer a convenient method for following the protein distribution of the various fractions during the isolation of the " purified " virus concentrate, it was necessary to determine how much of the protein in the particles took part in the colour reaction. To do this, samples of pure virus or particulate protein were prepared by dissolving the defatted particles in a minimum amount of NaOH and precipitating the protein by the addition of an equal volume of 10 per cent trichloracetic acid. The precipitated protein was washed once with water and used for total nitrogen determinations by the micro-Kjeldahl method and for " biuret " protein determinations. The various preparations gave similar results and an average value for the Spekker absorptiometer reading given by 1 mg. cell particulate protein was 0·167 compared with 0·150 for fowl serum protein. It was found that the biuret colour intensity was linear for amounts of protein less than 3·0 mg. per 2·0 ml. of biuret complex.

When estimating quantities of whole particulates in the " purified " fractions, a linear colour relationship existed for amounts corresponding to 0·05 to 5·25 mg. dry weight particulates per 2·0 ml. biuret complex.

Next, various preparations of cell particulates from normal fowl tissues and a non filterable fowl sarcoma (GRCH/16, Peacock and Peacock, 1953) and purified virus from Rous No. 1 sarcoma were subjected to (1) total nitrogen determinations by the micro-Kjeldahl method, (2) dry weight and (3) biuret protein determinations in order to estimate the ratio " Directly estimated protein nitrogen " : " Total nitrogen " (Table I).

TABLE I.—*Dry Weight, Total Nitrogen and Biuret Protein of Cell Particulates Deposited from Normal and Malignant Fowl Tissues by Centrifuging at* 12,000 *r.p.m. for* 55 *minutes.*

| Source. | Dry wt. mg./g. wet tissue. | Total N mg./g. wet tissue. | Biuret protein mg./g. wet tissue. | Total N / Dry wt. (%). | Protein / Dry wt. (%). | Protein N / Total N (%). |
|---|---|---|---|---|---|---|
| Rous No. 1 Sarcoma | 0·43 | 0·037 | 0·198 | 8·6 | 46 | 85 |
| ,, ,, * | — | 0·095 | 0·49 | — | — | 82 |
| ,, ,, * | — | 0·090 | 0·41 | — | — | 73 |
| GRCH/16 | 4·10 | 0·340 | 1·68 | 8·3 | 41 | 79 |
| Liver | 23·40 | 2·32 | 11·20 | 9·9 | 48 | 77 |
| Spleen | 5·10 | 0·414 | 1·99 | 8·1 | 39 | 78 |
| Brain | 2·94 | 0·241 | 1·15 | 8·2 | 39 | 76 |
| | | | Averages | 8·6 | 43±4 | 79±4 |

* Not referred back to original weight of tissue used. Figures indicate mg./ml. suspension.

The values of Total N : Dry weight give an average value of 8·6 per cent which agrees with the findings of other workers (Claude, 1938; Shemin and Sproule, 1942). The value of 79 per cent for the ratio protein N : Total N seems reasonable. It is not known exactly how much of the nitrogen in these particles can be attributed to protein since the amounts of nucleic acid are not accurately known. Claude (1939) estimated that 10–15 per cent of the protein fraction of Rous sarcoma virus (5–8 per cent of the total virus material) was nucleic acid using u.v. absorption methods, whereas Shemin and Sproule (1942), basing their estimation on purine nitrogen values, gave approximately 1·5 per cent of total virus material as nucleic acid. By using these values for nucleic acid and assuming lipid to be 50 per cent of the total material (with a nitrogen content of 1·6 per cent) it can be easily calculated that the protein nitrogen constitutes between 76 per cent and 87 per cent of the total N. The value of 79 per cent found in the foregoing experiments lies between the calculated figures and it thus seems that most, if not all the particulate and virus protein enters into the biuret reaction.

(2) *Protein distribution during virus purification procedure.*

The biuret method was used, therefore, to follow the protein distribution during the purification of the Rous sarcoma virus. At the same time, the virus activity of each fraction was determined by titration in day old chicks. In one experiment 26·5 g. of fresh tumour tissue were processed according to the diagram shown in Fig. 1 and the results of the experiment are given in Table II.

All the fractions were tested for activity except $D_2B$ (second deposit after trypsin digestion). There is usually some aggregated material present in this

TABLE II.—*Protein Distribution at each each Stage of the Extraction of 26·5g. Rous Sarcoma Tissue.*

| Fraction. | Mg. protein in 26·5 g. tumour. | Protein as % total protein. | Dilution necessary for tumour production × 0·2 c.c. | Mg. protein in 0·2 c.c. | Mg. protein required for infection. |
|---|---|---|---|---|---|
| $S_1$ | 460 | — | — | — | — |
| $S_2$ | 430 | 94 | $10^{-4}$ | ·33 | $3·3\times10^{-5}$ |
| $D_2A$ | 38 | 8·3 | $10^{-5}$ | ·38 | $3·8\times10^{-6}$ |
| $D_2B$ (trypsin) | 33 | 7·2 | — | — | — |
| $S_3$ | 26 | 5·6 | $10^{-5}$ | ·20 | $2·0\times10^{-6}$ |
| $D_3$ | 4·2 | ·91 | $10^{-2}$ | ·13 | $1·3\times10^{-3}$ |
| $S_4$ | 14·6 | 3·2 | $10^{-3}$ | ·11 | $1·1\times10^{-4}$ |
| $D_4$ | 6·3 | 1·4 | $10^{-5}$ | ·02 | $2·0\times10^{-7}$ |

fraction which does not form a stable suspension and any attempt to titrate it gives rise to unreliable results. The figures in Column 1 of Table II are direct measurements of protein and some discrepancies in the amounts at each stage are unavoidable but the final column shows that the virus concentrate, $D_4$, provides the maximum infectivity with the least amount of protein. $2·0 \times 10^{-7}$ g. compares favourably with the results of other workers. Treatment with the enzyme trypsin has removed 19·6 mg. "biuret" protein from 38 mg. in $D_2A$ (52 per cent) which is close to the value of 60 per cent found by Carr and Harris (1951). It is also apparent that the final virus concentrate ($D_4$) contains approximately 1·4 per cent of the protein in the original extract yet retains most of the infectivity. This, again, agrees closely with results using nitrogen estimations.

(3) *Effect of trypsin treatment on the microscopic appearance of extracts of Rous No.* 1 *sarcoma.*

Samples of the partially purified extracts at Stages $D_2A$, $D_2B$ and $S_3$ (Fig. 1) were examined in the dark field microscope in order to ensure that the "purified" virus concentrate was free from non particulate material. The examination showed that $D_2A$ (resuspended deposit from first high speed run) consisted of fairly coarse aggregates, some filaments and large spheres and many smaller particulates. $D_2B$ (same after trypsin treatment) contained a larger proportion of small particulates and few filaments or large spheres. $S_3$ (after clarification at 3000 r.p.m.) showed a homogenous suspension of fine particulates with very few of the larger bodies present.

(4) *Virus particle estimation and infectivity titrations for Rous No.* 1 *sarcomata growing in hosts of various ages.*

The chickens were inoculated with a 10 per cent suspension of Rous sarcoma cells (0·5 ml.) in both pectoral muscles and the size of the tumours was determined by palpation. All extractions were made of tumours between 12 and 28 days of age and usually between 14 and 21 days. The + sign method for estimating size was used, + being just palpable, ++ covering one quarter of the breast, +++ one half and ++++ the entire breast. For convenience, only two groups have been used in the table of results, (Table III) slow growing tumours being considered as all those less than +++ in size at the time of extraction. The virus particle measurements and infectivity titrations were done by the methods described in the first part of the paper.

The results include 35 extractions and cover a wide range of infectivity values. The final column in Tables III and IV (virus yields) represent the dry weight of whole virus particles (including the lipoid portion) as determined by the biuret

TABLE III.—" *Purified* " *Virus Content and Infectivity Titres of Fast and Slow Growing Rous No.* 1 *Sarcomata from Hosts of Various Ages.*

| Age of host (days). | Infectivity (M.I.D./g. wet tumour). Fast growing. | Slow growing. | " Purified " virus content mg./g. wet tumour. |
|---|---|---|---|
| 22 | $10^7$ | — | ·49 |
| 28 | — | $10^5$ | ·37 |
| 32 | $10^6$ | — | ·25 |
| 51 | $10^5$ | — | ·33 |
| 56 | $10^5$ | — | ·24 |
| 57 | $10^6$ | — | ·43 |
| 57 | — | $10^4$ | ·52 |
| 58 | $10^6$ | — | ·60 |
| 65 | $1C^6$ | — | ·43 |
| 66 | $10^5$ | — | ·28 |
| 66 | $10^4$ | — | ·36 |
| 70 | — | $10^3$ | ·45 |
| 72 | — | $10^4$ | ·70 |
| 73 | $10^5$ | — | ·34 |
| 78 | $10^5$ | — | ·27 |
| 78 | $10^6$ | — | ·31 |
| 82 | $10^6$ | — | ·28 |
| 84 | $10^6$ | — | ·51 |
| 87 | — | $10^3$ | ·36 |
| 91 | — | $10^3$ | ·56 |
| 105 | $10^5$ | — | ·36 |
| 108 | $10^5$ | — | ·38 |
| 115 | $10^5$ | — | ·52 |
| 143 | — | $<10^2$ | ·38 |
| 143 | — | $<10^2$ | ·59 |
| 161 | $10^5$ | — | ·40 |
| 161 | $10^5$ | — | ·29 |
| 192* | $>10^4$ | — | ·38 |
| 213* | $>10^4$ | — | ·34 |
| 213 | — | $<10^2$ | ·43 |
| 442 | — | $10^4$ | ·88 |
| 446 | $10^4$ | — | ·44 |
| 447 | $10^5$ | — | ·52 |
| 454 | — | $10^5$ | ·68 |
| 253† | — | $<10$ | ·43 |
| | | Average | ·46 ± ·14 |

* The extracts from these two birds were not diluted sufficiently to reach a true end point, but it was apparent from the latent period of the tumours appearing in the chicks treated with the $10^{-4}$ dilution that at least a further 1/10 dilution would have produced a response.

† This bird developed a " recurring tumour " (Carr, 1942) which appeared 150 days after the original inoculation in a chicken of the " Non-susceptible " strain.

TABLE IV.—*Average Virus Concentrate Yields arranged in order of Increasing Virus Infectivity.*

| M.I.D./g. tumour. | No. of observations. | " Purified " virus yield and standard deviation. |
|---|---|---|
| $10^3$ or less | 7 | ·46 ± ·09 |
| $10^4$ and $10^5$ | 20 | ·43 ± ·16 |
| $10^6$ and over | 8 | ·41 ± ·12 |

procedure. The recent experiment of Duran-Reynals and Freire (1953) in which tumours growing in old hosts exhibited a larger number of non-infective filtrates is borne out in this work by the fact that the only non-infective extracts encountered occurred in hosts over 140 days old (Table III). The data also include five pairs of chickens which can be compared as regards infectivity of slow and fast growing tumours of the same age. The members of each pair came from the same hatch and were inoculated at the same time with similar amounts of Rous cell suspensions. In every case the slow growing tumour contained less infective virus than the fast growing one although the average yields of virus material were almost identical, namely 0·43 mg./g. from fast growing and 0·46 mg./g. from slow growing tumours. The observations of Carr (1953) that slow growing tumours contain less *infective* virus than fast growing ones are, therefore, confirmed in this series. In Table IV, the results are arranged in groups of increasing infectivity. Analysis of variance showed that there was no significant difference between the " purified " virus yields of any of the groups at the 5 per cent level or 10 per cent level although the infective capacity of the preparations ranged from 0 to $10^7$ minimum infective doses (M.I.D.) per g. of tumour tissue.

### DISCUSSION.

The preliminary experiments show that it is possible to adapt the biuret reaction for proteins to the quantitative estimation of cytoplasmic particles and viruses provided the large proportion of fat associated with them is removed by shaking with ether or by some similar procedure. This technique should prove useful under a variety of conditions as it is fairly certain that all of the protein in the particles is involved in the reaction. The fact that the biuret method yields similar results to those obtained with the micro-Kjeldahl nitrogen procedure when applied to the distribution of protein during purification of Rous sarcoma virus shows that the method, though more quickly and easily done, yields reliable results. It is apparent from the figures in Tables III and IV that, within the limits of the experimental conditions, no very marked differences in the amounts of " purified " virus material could be detected despite the wide variation in infectivity. This is in agreement with the observations of Eckert *et al* (1952) on the virus particle content of the plasma of birds with erythromyeloblastic leukosis. These authors could find no correlation between the infectivity, number of primitive cells, or the number of virus particles deposited by ultra centrifugation and counted directly in the electron microscope.

Such observations, of course, raise the question of the purity of the preparations used. In the present work, the method of virus purification is one which has been selected as giving the highest activity with the least expenditure of time so that virus will not be lost through oxidation and excessive handling. Since it has been shown that concentrated tumour extracts are almost completely agglutinated by fowl virus-neutralizing antibody (Amies, 1937) and that anti-fowl protein precipitins were only present in very low concentrations in the sera of rabbits sensitized to tumour virus particles (Amies and Carr, 1939) it seems reasonable to assume that the " purified " virus concentrates are related to Rous sarcoma tissue and foreign to fowl tissue. Such studies as these combined with the fact that the agent behaves as a single, homogeneous substance electrophoretically (Shemin and Sproule, 1942) seem to indicate that the large number of particles necessary for infection is due to most of the material being composed of "inactive virus " and bring the results for the Rous agent into line with other viruses. If the mass of a Rous virus particle is taken as $3 \cdot 2 \times 10^{-16}$ g. (calculated for a sphere of radius $4 \cdot 0 \times 10^{-6}$ cm. and a density of 1·3), then the extracts employed here contain approximately $1 \cdot 4 \times 10^{12}$ particles per gram of tumour tissue. The most active tumour yielded $10^7$ infective doses/g., therefore the number of particles required to initiate a tumour response in this case was of the order of 100,000. Claude (1939) and Claude and Rothen (1942) using similar techniques were able to prepare an extract in which only about 2000 particles were necessary for infection, but such differences may be due to variation in the chicken strains. The work of Eckert *et al.* (1952), already mentioned, has shown that in erythromyeloblastic leukosis, the infectivity of the plasmas exhibited a range of at least five $\log_{10}$ increments, while the numbers of particles only varied from $0 \cdot 1 \times 10^9$ to $7 \cdot 2 \times 10^9$ per ml. Bryan (1941) showed that for Shope papilloma virus, $9 \cdot 4 \times 10^7$ particles were necessary for a 50 per cent infectious unit. Both of these viruses show evidence of existence in a " masked " form, as does Rous No.1 agent.

The production of large amounts of non-infective material is not confined to tumour viruses alone, and has been observed in other types, whether plant, animal or bacterial. Bawden and Pirie (1946) estimated that at least $10^7$ particles of tobacco mosiac virus were necessary to provide an infective dose and were able to separate their preparations into fractions with different infectivities. Gard and von Magnus (1946) showed that large doses of influenza virus cause the cells lining the allantoic cavity of eggs to produce non-infective agglutinin and Doermann (1951) demonstrated the multiplication of $T_2$ bacteriophages in a non-infective form by releasing them at various times during their growth cycle. These and many other observations lead to the conclusion that infectivity is one of the final properties with which a virus particle becomes endowed. The nature of the tumour virus growth cycle, which, after the initial infection, remains entirely intracellular, probably favours the very large production of non-infective material which has been observed in these experiments.

It would appear then, that, any changes in the virus must be of quality rather than quantity, i.e., an alteration of the proportions of infective to non-infective virus. That the non-infective material is still capable of causing tumour growth is apparent from the many observations on " masked virus " which shows no infectivity but which later becomes capable of infecting when the tumour is transplanted to new hosts. It is possible that the ratio, infective : non-infective virus may give rise to larger variations in infectivity than would be expected from simply a larger proportion of the infective phase, due to the phenomenon of interference already noted in some animal and plant viruses and bacteriophages (Henle, 1950 ; Luria and Delbruck, 1942 ; Kleczkowski and Kleczkowski, 1953,; Bawden and Kleczkowski, 1953), In the case of Rous No. 1 sarcoma the most important factors influencing the ratio of the two phases seems to be reflected in the rate of growth of the tumour and the age of the host. While it is true that the infectivity of Rous No. 1 sarcomata is inversely proportional to the age of the tumour itself, this effect is caused by an increase in circulating antibodies (Carr, 1943). The antibodies reduce the amount of infective virus by forming floccules with virus which are lost during the extraction procedure, and by neutralizing the virus remaining in suspension. When tumours 12 to 28 days of age are used, as in this work, the antibodies have little or no effect on the infectivity of the extracts. The reduced amounts of infective virus found in old hosts by Carr (1953)

while not so strikingly evident in this series of birds, would appear to be borne out by the fact that $10^7$ M.I.D./g. were only encountered once, in a very young host, and $10^6$ M.I.D./g. were never observed in extracts from hosts over 100 days old. Old hosts did, however, produce a larger number of slow growing tumours showing little or no necrosis and haemorrhage and yielding less infective virus, and the only tumours yielding non-infective filtrates were found in hosts over 140 days old.

SUMMARY

1. The biuret reaction for proteins has been adapted for use in making estimates of the yields of cytoplasmic particles, with which virus activity is associated, from Rous No. 1 sarcomata. The method gives results comparable to those obtained with total nitrogen estimations and it is fairly certain that all the protein contained in the particles is involved in the reaction.

2. Measurements of purified virus yields and virus infectivity of extracts of 35 tumours of different growth rates in hosts of various ages show that although infectivity varies from 0 to $10^7$ minimum infective doses per g. of tumour, the virus yields remain fairly constant at $0{\cdot}46 \pm 0{\cdot}14$ mg./g. ($1{\cdot}4 \times 10^{12}$ particles/g.). No significant differences appear when the extracts are divided into groups of low, medium and high infectivity. It is concluded that the differences in infectivity are probably due to qualitative alterations in the ratio, infective : non-infective virus, rather than a quantitative change.

3. The observations support the recent finding of reduced infectivity in tumours of adult hosts and in slow growing tumours. The reduced occurrence of non-infective tumours in old hosts has also been confirmed.

All expenses in connection with this work were borne by the British Empire Cancer Campaign.

REFERENCES.

Amies, C. R.—(1937) *J. Path. Bact.*, **44**, 141.
*Idem* and Carr, J. G.—(1939) *Ibid.*, **49**, 497.
Bawden, F. C., and Kleczkowski, A.—(1953) *J. gen. Microbiol.*, **8**, 145.
*Idem* and Pirie, N. W.—(1946) *Brit. J. exp. Path.*, **27**, 81.
Bryan, W. Ray—(1941) *J. nat. Cancer Inst.*, **1**, 607.
Carr, J. G.—(1942) *Brit. J. exp. Path.*, **23**, 206.—(1943) *Ibid.*, **24**, 133.—(1953) 'Symposium on the Nature of Virus Multiplication.' Oxford (Cambridge University Press), p. 284.
*Idem* and Harris, R. J. C.—(1951) *Brit. J. Cancer*, **5**, 83.
Claude, A.—(1938) *Science*, **87**, 467.—(1939) *Ibid.*, **90**, 213.
*Idem* and Rothen, A.—(1942) *J. exp. Med.*, **71**, 639.
Doerman, A. H.—(1951) *Fed. Proc.*, **10**, 591.
Duran-Reynals, F., and Freire, P. M.—(1953) *Cancer Res.*, **13**, 376.
Eckart, E. A., Sharp, D. G., Beard, Dorothy and Beard, J. W.—(1952) *J. nat. Cancer Inst.*, **13**, 533.
Gard, S., and von Magnus, P.—(1946) *Ark. Kemi Min. Geol.*, **24b**, No. 8.
Henle, W.—(1950) *J. Immunol.*, **64**, 203.
Kleczkowski, J., and Kleczkowski, A.—(1953) *J. gen. Microbiol.*, **8**, 135.
Luria, S. E., and Delbruck, M.—(1942) *Arch. Biochem.*, **1**, 207.
Peacock, P. R., and Peacock, Andree—(1953) *Brit. J. Cancer*, **7**, 120.
Shemin, D., and Sproule, E. E.—(1942) *Cancer Res.*, **2**, 514.
Stickland, L. H.—(1951) *J. gen. Microbiol.*, **5**, 698.
Weichselbaum, T. E.—(1946) *Amer. J. clin. Path.* (Tech. Sec.), **16**, 40.